# Development of Antibody Therapeutics against Flaviviruses

**DOI:** 10.3390/ijms19010054

**Published:** 2017-12-25

**Authors:** Haiyan Sun, Qiang Chen, Huafang Lai

**Affiliations:** The Biodesign Institute, School of Life Sciences, Arizona State University, 1001 S. McAllister Avenue, Tempe, AZ 85287, USA; haiyan.sun@asu.edu (H.S.); Huafang.lai@asu.edu (H.L.)

**Keywords:** flavivirus, antibody, monoclonal antibody (mAb), therapeutics, plant-made antibody, antibody-dependent enhancement (ADE), West Nile virus (WNV), Dengue virus (DENV), Zika virus (ZIKV), plant-made pharmaceuticals (PMP)

## Abstract

Recent outbreaks of Zika virus (ZIKV) highlight the urgent need to develop efficacious interventions against flaviviruses, many of which cause devastating epidemics around the world. Monoclonal antibodies (mAb) have been at the forefront of treatment for cancer and a wide array of other diseases due to their specificity and potency. While mammalian cell-produced mAbs have shown promise as therapeutic candidates against several flaviviruses, their eventual approval for human application still faces several challenges including their potential risk of predisposing treated patients to more severe secondary infection by a heterologous flavivirus through antibody-dependent enhancement (ADE). The high cost associated with mAb production in mammalian cell cultures also poses a challenge for the feasible application of these drugs to the developing world where the majority of flavivirus infection occurs. Here, we review the current therapeutic mAb candidates against various flaviviruses including West Nile (WNV), Dengue virus (DENV), and ZIKV. The progress of using plants for developing safer and more economical mAb therapeutics against flaviviruses is discussed within the context of their expression, characterization, downstream processing, neutralization, and in vivo efficacy. The progress of using plant glycoengineering to address ADE, the major impediment of flavivirus therapeutic development, is highlighted. These advancements suggest that plant-based systems are excellent alternatives for addressing the remaining challenges of mAb therapeutic development against flavivirus and may facilitate the eventual commercialization of these drug candidates.

## 1. Introduction

The *Flavivirus* genus belongs to the family of *Flaviviridae*, which consists of more than 70 viruses including insect-specific flaviviruses [1,2,3,4]. The majority of flaviviruses are transmitted by mosquitos or ticks [5]. Many flaviviruses are important human pathogens, including yellow fever virus (YFV), four serotypes of Dengue viruses (DENV1–4), West Nile virus (WNV), Japanese encephalitis virus (JEV), tick-borne encephalitis virus (TBEV), and, recently, Zika virus (ZIKV). While some flavivirus infections are either asymptomatic or cause mild symptoms, others are causative agents of serious human diseases including hemorrhagic fever, encephalitis, meningitis, and other severe neurological complications. Some of the flaviviruses can also persist in patients to cause long-term morbidities [6,7]. Flavivirus infections have become a global public health burden. It was estimated that close to 400 million people worldwide are at risk of being infected with DENV each year [8,9]. In 2016, an YFV outbreak in Africa infected more than 7000 people and caused nearly 400 deaths [10]. The unexpected outbreak of ZIKV in Latin America led to the declaration of a global public health emergency by the World Health Organization (WHO) in 2016 because of the link between ZIKV infection and diseases of the central nervous system (CNS) including microcephaly and Guillain–Barré syndrome [11]. Currently, there are no licensed therapeutics for flavivirus infection; all treatment options are directed at reducing fluid loss or inflammation caused by viral infection [5]. Although vaccines based on inactivated or attenuated viruses against YFV, TBEV, JEV, and recently DENV have been licensed for human use, outbreaks of these flaviviruses still occur despite the availability of these vaccines [12,13]. This exposes the difficulty of implementing successful vaccination programs and highlights the urgency of developing therapeutics.

**Figure 1:**
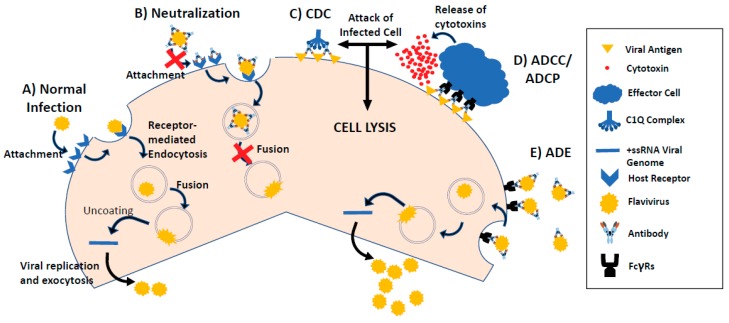
Flavivirus infection cycle and mechanisms of mAb neutralization and enhancement. The entry of flaviviruses into host cells is initiated with the attachment of the E protein with its receptor on the target host cell, which leads to endocytosis of flavivirus virions (**A**). The low pH in the endosome triggers the fusion of the viral envelope with the endosomal membrane, releasing the viral genome to the cytoplasm where viral replication and assembly occur (**A**). MAbs can neutralize flaviviruses by blocking viral attachment, endocytosis, or membrane fusion (**B**). MAbs can eliminate flavivirus-infected cells through antibody Fc effector functions such as complement dependent cytotoxicity (CDC) (**C**) and antibody-dependent cell cytotoxicity (ADCC) (**D**). Some non-neutralizing or subneutralizing anti-flavivirus mAbs can enhance viral infection in Fc receptor-expressing cells via the mechanism of antibody-dependent enhancement (ADE) (**E**).

Flaviviruses are positive single-stranded RNA viruses with a genome size of ~11 kb. The viral genome encodes 10 viral proteins including three structural proteins (capsid, membrane and envelope proteins) and seven non-structural proteins (NS1, NS2A, NS2B, NS3, NS4A, NS4B, NS5). The mature flavivirus virion shares similar structure with icosahedral symmetry, with the surface covered by 180 copies of envelope (E) glycoproteins and membrane (M) proteins and a less organized nucleocapsid core consists of multiple copies of capsid protein (C) and the RNA genome [14,15,16,17]. The entry of flaviviruses into host cells relies on the proper contact of the E protein with its receptor on the target host cell. Studies from DENV and other flaviviruses show that the E protein does not directly bind to its receptor on the first contact with the target cell. Instead, the E protein binds first to attachment factors such as glycosaminoglycans (GAGs) on the host cell surface. Such binding enriches the density of viral particles on the cell surface and leads to the high-affinity interaction between the E protein and its target receptor [18]. Many cellular receptors have been reported for flavivirus entry into different cell types, such as C-type lectin receptors, laminin receptor, T-cell immunoglobulin and mucin domain (TIM) and TYRO3, AXL and MER (TAM) receptors, and integrin αvβ3 [18]. Binding to these receptors has been shown to lead to the low pH dependent endocytosis of flavivirus virions (Figure 1A). However, the complete flavivirus entry pathway is still not fully understood and the cellular components absolutely required for flavivirus entry have not been completely identified [19]. In the endosomes, the viral envelope fuses with the host membrane and releases the viral capsids into the cytoplasm where viral protein translation, RNA genome replication and virus particle assembly take place [20] (Figure 1A). Advancement in the understanding of the life cycle of flaviviruses facilitates the development of therapeutics to target various stages of viral pathogenesis.

## 2. Monoclonal Antibody as Therapy

Since the breakthrough of monoclonal antibody (mAb) production using hybridoma cell culture in 1975, mAbs have become one of the major targets for drug discovery [21]. As of 2017, approximately 70 mAbs have been approved by the United States Food and Drug Administration (FDA) for treating a variety of human diseases including cancers, chronic inflammatory diseases, infectious diseases and neurodegenerative diseases [22].

For cancer treatment, mAbs have been found to kill cancer cells through several mechanisms [23]. Many mAbs directly target the tumor-associated antigens on the cell surface. Such mAb-tumor antigen interactions can activate or block certain downstream signaling pathways and, in turn, reduce tumor cell proliferation or induce tumor cell apoptosis [24,25]. However, increasing evidence suggests that mAbs also kill tumor cells through activation of host immune responses such as phagocytosis, complement dependent cytotoxicity (CDC), or antibody-dependent cell cytotoxicity (ADCC) [23]. ADCC is exerted by immune cells expressing surface Fc gamma receptors (FcγRs) against cells coated with antibody, such as cancer or virus-infected cells (Figure 1). The typical ADCC involves activation of natural killer (NK) cells by antibodies. FcγRs expressed on the surface of NK cells recognize the Fc portion of an antibody, which has bound to the surface of a cancer or pathogen-infected target cell. Once the FcγR binds to the Fc region of an antibody, the NK cell releases cytotoxic factors that cause the lysis of cancer or infected target cells [26] (Figure 1). In contrast, CDC is executed by a protein-based system called complement that attacks target cells bound with antibodies. CDC is triggered by the binding of C1q, a component of the complement, to the Fc region of an antibody that is attached to the target cell. This binding activates the complement cascades, leading to the formation of the membrane attack complex at the surface of the target cell, triggering target cell lysis [26] (Figure 1). Activation of antibody effector functions as a major mechanism of action has been demonstrated by many therapeutic mAbs including rituximab, trastuzumab and cetuximab. For example, Rituximab targets CD20 of CD20-positive B-cell lymphomas and has been shown to kill cancer cells by its potent CDC and ADCC activities [27,28]. It has also been shown that breast cancer patients who responded with remission to Her2/neu-specific trastuzumab have a higher capacity to mediate ADCC than patients who failed to respond to trastuzumab treatment [29]. Studies also suggest that ADCC activity is one of the modes of therapeutic action of cetuximab against colorectal cancer that targets the epidermal growth factor receptor (EGFR) [30]. These results highlight the key role of activation of immune functions in anti-tumor effects by mAb-based therapeutics.

Infectious diseases are another area that mAb-based therapies have shown promising results. Using mAbs to treat infectious diseases is inspired by the limited success of convalescent therapy with convalescent whole blood (CWB) or convalescent plasma (CP) in treating viral infections such as influenza, measles, and coronaviruses [31]. These treatments helped to improve symptoms and reduce mortality when other treatment options were not available especially during an epidemic outbreak. More recently, CWB was used to treat Ebola virus-infected patients during the 2014–2015 Ebola outbreak in West Africa [32]. However, the efficacy and safety of using convalescent blood products as therapeutics have not been fully evaluated [33]. The risk of transmitting pathogens via transfusion, the need of extensive screening of blood donors, and the pathogen elimination process further hinder the broad application of this strategy. Currently, there are nearly 40 mAbs in clinical development worldwide, targeting more than a dozen infectious diseases including human immunodeficiency virus (HIV), anthrax, Ebola, hepatitis, and influenza [34]. Three mAbs have been licensed for the treatment or prevention of infectious diseases and are in clinical use [34]. Palivizumab was approved by FDA in 1998 for the prevention of respiratory syncytial virus (RSV) infection, which causes serious symptoms in the lower respiratory tract of infants and young children [35]. In vitro studies showed that palivizumab binds to the F glycoprotein of RSV, thereby blocking the viral fusion with host cell membrane [36,37]. Raxibacumab and obiltoxaximab are licensed to treat inhalational anthrax by directly preventing the binding of bacterial antigens to their respective cellular receptors [38]. Multiple mAbs against HIV has been developed and several of them have made their way into human clinical trials. For example, mAbs against the HIV fusion co-receptor CCR5 have been shown to broadly and potently inhibit HIV-1 in vitro by occluding HIV’s access to CCR5 and preventing membrane fusion [39]. Furthermore, their potent antiviral activity has been demonstrated in HIV-infected individuals in clinical trials [39]. Recently, ibalizumab, a mAb that blocks viral entry into host cells has shown efficacy for patients with drug-resist HIV in a phase 3 human trial [40]. Both in vitro and in vivo studies with HIV Env-specific mAbs such as b12, PGT121, and 10–1074 demonstrated that ADCC activity is an important mechanism of mAbs in treating viral infection, especially those caused by cell-associated HIV [41]. These studies illustrated that mAbs are promising therapeutics in treating infectious diseases that can eliminate pathogen infection through multiple mechanisms including neutralization to block viral attachment and fusion, and antibody-induced effector functions (Figure 1).

Compared to small molecule drugs, mAb-based therapeutics have several unique advantages. For example, mAbs are highly specific and human or humanized mAbs generally have lower immunogenicity or off-target toxicity. MAbs act through multiple mechanisms including direct targeting of specific antigens to block pathogen attachment, prevent fusion with host membranes, and modulate effector functions (i.e., ADCC, CDC) (Figure 1). Moreover, mAb therapy can be used in combination with other traditional therapeutics, which has been shown to offer additive benefits over individual therapies without additional toxicity [42,43]. As a result, mAb-based therapeutics are promising candidates in treating infectious diseases, especially for those exhibiting multidrug resistance (MDR) [44]. However, mAb therapeutics are slow to develop and expensive to produce, rendering them affordable only for citizens in a few developed countries [45].

## 3. Potential Therapeutic Antibodies against Flavivirus

MAbs can protect against flavivirus infection at multiple steps during the virus entry such as blocking virus attachment to the cell surface, interrupting viral membrane fusion, or activating Fc-dependent effector functions [46] (Figure 1). However, weak or neutralizing antibodies at sub-neutralizing concentrations may cause antibody-dependent enhancement (ADE) of infection, a phenomenon most often associated with DENV [47]. ADE occurs when non-neutralizing antibodies or antibodies at sub-neutralizing concentrations bind to both the virus and FcγR on the cell surface, thereby facilitating virus entry and increases the infection rate (Figure 1) (see Section 4).

**Figure 2:**
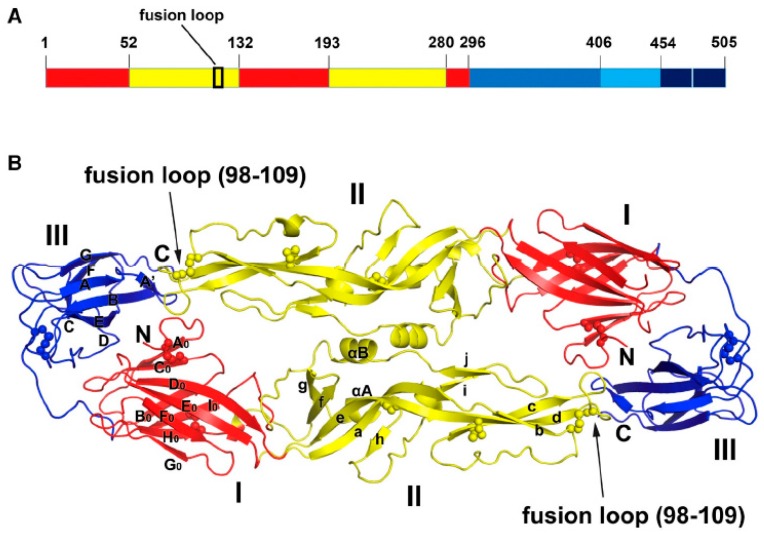
Structure of the E protein of ZIKV. (**A**) Domain organization of ZIKV E protein. Domain I, II and III are schematically indicated with red, yellow, and blue bars, respectively. (**B**) Dimer structure of the E protein of ZIKV. Domain I, II and III follow the same color scheme as Panel A and the position of fusion loop is indicated with an arrow. The locations of epitopes for various antibodies are indicated with spheres. (From [54] with permission from Elsevier).

Most neutralizing antibodies against flaviviruses identified so far are found to target the E protein [48,49] (Table 1). Structural studies of the E protein of several flaviviruses have revealed that it shares a common three-domain architecture among several important flaviviruses (Figure 2) [50,51,52,53,54]. E protein domain I (DI) is an eight-stranded β barrel located at the center of the E protein, while domain II (DII) consists of two long finger-like structures with a highly conserved 13-amino acid fusion loop that is responsible for the membrane fusion during viral entry. Domain III (DIII) is an immunoglobulin-like structure at the C-terminus of the E protein. It has been suggested to play a critical role in receptor recognition during virus attachment. Antibodies isolated from WNV, DENV, or ZIKV-infected human or mouse sera have been mapped to all three domains of E protein. In addition to the E protein, protective antibodies against other flavivirus proteins have also been characterized. For example, antibodies against M and NS1 protein have been shown to protect mice against lethal DENV or WNV infections, respectively [55,56]. However, most antibodies against non-E proteins are typically non-neutralizing, which make them less likely to be considered as candidates for therapeutics. Thus, in this review we will focus on antibodies against the E protein.

### 3.1. Antibodies Targeting DIII

To date, the most potent neutralizing antibodies against flaviviruses are mapped to domain III of the E protein [8,57,58,59]. Antibodies mapped to DIII are typically virus or serotype-specific and less cross reactive, perhaps due to the lowest sequence identity between different flaviviruses among the three domains of the E protein [58]. Several important epitopes in DIII have been identified through panning of memory B cells collected from either flavivirus-infected patients or challenged mice. These epitopes include the lateral ridge [57,60,61], C-C′ loop [61,62] and A strand [59,63] in DIII.

The first well characterized anti-DIII mAb with strong therapeutic potential was the anti-WNV E16 [57,64]. This mAb was originally isolated from mice immunized with a recombinant WNV E protein [57]. In vitro neutralization assays showed that E16 and humanized E16 (hE16) neutralize WNV infection at 10–20 nanomolar (nM) concentrations. Post-exposure therapeutic studies revealed that one single-dose injection protected mice from a lethal WNV challenge [57]. Crystal structure of the E16 antigen-binding fragment (Fab) and the WNV E protein DIII complex revealed that E16 has contact with 16 amino acid residues from four discontinuous segments located in the *N*-terminal region and three strand connecting loops (BC, DE, FG) of DIII, respectively; together they form a surface patch on the lateral ridge of DIII [57]. Results from studies of a cryo-EM structure of the E16 Fab-WNV complex and in vitro assays suggest that E16 primarily inhibits viral entry at a post-attachment step, probably through interrupting the conformational rearrangement of E protein before membrane fusion [57,64]. Studies with E16 Fc mutants or FcγR-mutant mice indicated that E16 also control WNV infection through ADCC and C1q-related effector functions (Figure 1) [65]. In light of its superior preclinical results, hE16 entered a Phase II human clinical trial in 2009 [66]. However, the trial was eventually terminated due to the difficulty of recruiting a sufficient number of WNV-infected patients with the required clinical symptoms during the trial period [67].

Two DENV1 specific mAbs were also mapped to the lateral ridge of DIII [68,69]. DENV1-E105 and E106 showed strong post-exposure protection in an immunocompromised mouse model [69]. Even though both mAbs neutralize all DENV1 genotypes in 3–4 nM ranges, they cannot neutralize other serotypes of DENV [68,69], making them likely suspects of inducing ADE upon secondary infection by other DENV serotypes (see Section 4 below).

Due to the recent outbreaks of ZIKV, antibodies against this flavivirus have been intensively investigated in search of effective therapeutics. Several highly potent mAbs have been mapped to the lateral ridge of DIII. ZV54 and ZV67 are mAbs isolated from mice immunized with live ZIKV [61]. The antibodies neutralized all four tested ZIKV strains and passive transfer of these two mAbs protected mice from a ZIKV lethal challenge in an interferon γ deficient mouse model. Crystal structure of the ZV67-ZIKV DIII complex suggests that ZV54 and ZV67, which only differ in two contact residues, bind to ZIKV DIII lateral ridge very similarly to WNV E16 or DENV1-E106 [61]. This indicates that the DIII lateral ridge epitopes are highly conserved among different flaviviruses. Interestingly, Z004, a mAb that potently neutralizes both ZIKV and DENV1 also recognizes the lateral ridge of ZIKV or DENV1 DIII [60].

Antibodies recognizing the C-C′ loop in DIII were first described in a structural and functional study of a pool of antibodies generated by mice infected with a mixture of two DENV2 strains [62]. These antibodies showed different potency for neutralizing various strains of DENV2 with protective effect only for specific strains. Further studies suggest that they inhibit DENV2 infection at a post-attachment step. Consistent with the findings from DENV2, crystal structures of a DENV1 antibody (DENV1-E111) bound to the C-C′ loop of DIII of two different genotypes of DENV1 reveal that the potency of neutralization may rely on the extent of exposure of the C-C′ loop in DIII of a particular genotype [70]. More recently, two anti-ZIKV mAbs targeting the C-C′ loop (ZV48 and ZV64) were found to neutralize only two of the four tested ZIKV strains [61]. In contrast, the two mAbs targeting the lateral ridge of DIII (ZV54 and ZV67) could neutralize all four tested strains of ZIKV [61]. These results indicate that antibodies recognize the C-C′ loop have less predictable potencies than those recognizing the lateral ridge of DIII.

DENV E protein DIII β-strand A has been shown as an important region for antibody binding from early studies [71,72,73]. Antibodies mapped to this region have shown potent neutralization of some DENV serotypes, but not to all four serotypes [62,63,74]. Structural studies of two such mAbs (1A1D-2 and 4E11) complexed with DENV E proteins indicate that the antibody probably binds to the A-strand epitope during a “breathing” state of the virion, thereby disrupting the mature virion architecture and preventing its binding to cell surface receptors [74,75]. Using a computational chemistry approach, Tharakaraman et al. redesigned the 4E11 antibody by a combination of five affinity-enhancing mutations; the resulting antibody, 4E5A, showed a 450-fold increase in DENV4 binding affinity while retaining potent affinity to DENV1–3 [63]. Consistent with the results of binding affinity, this mAb demonstrated a strong neutralization potency to all four DENV serotypes. With a similar approach, a humanized version of 4E5A was developed with six replacement and one deletion mutations [59]. The resultant mAb, named ab513, demonstrated strong neutralizing potency against all four serotypes of DENV, even in FcγR-mediated phagocytosis. In multiple mouse models of DENV infection, ab513 exhibits strong therapeutic effects against all four DENV serotypes, demonstrating its potential as an effective therapeutic agent in humans. Ab513 is currently under development by Visterra Inc. (Cambridge, MA, USA) and is expected to enter Phase I clinical trials by 2018 [76].

### 3.2. Antibodies Targeting DI, DII

Although E DIII contains the epitopes for the most potent and serotype-specific neutralizing antibodies, analysis of sera from flavivirus-infected human patients showed that antibodies mapped to DIII only account for a very small portion of the total E protein specific mAbs [77,78,79]. In contrast, the majority of E protein-specific antibodies from infected human sera were mapped to DI and DII of the E protein. In general, these anti-DI, DII antibodies are less potent in their neutralizing activity but are more cross-reactive among different serotypes/strains of various flaviviruses than those against DIII [49,77,79,80]. Several DI-DII epitopes have been identified, such as the DI-DII hinge, the highly conserved fusion loop of DII and the BC loop of DII [54,77,79,81,82,83,84,85].

The most commonly recognized epitope within the DI and DII region is probably the fusion loop epitope (FLE). FLE antibodies and their interactions with the E proteins have been well characterized [49,54,79,86,87,88]. They account for a significant portion of E protein-specific antibodies isolated from flavivirus-infected human sera [49,79,89,90]. FLE antibodies are cross-reactive and, therefore, may have both neutralizing and enhancing activities. The risk of enhancing heterologous flavivirus infection typically prevents FLE antibodies from being considered as therapeutic candidates. However, one of the FLE mAbs, 2A10G6, has been shown to bind to a DRXW motif within the fusion loop and has a broad neutralizing capability against all four serotypes of DENV, as well as YFV, TBEV, and WNV [84]. In addition, this mAb also demonstrated its therapeutic potency against lethal challenges of DENV and WNV in multiple mouse models [84]. More recently, its activity in high-affinity binding to the E protein and potency in neutralization and protection against lethal challenges of infection in mice has also been extended to ZIKV [54]. These results indicate that the highly conserved fusion loop region may contain epitopes for broadly neutralizing anti-flavivirus antibodies with therapeutic potential. In addition, FcγR- and complement-mediated pathways may play a role in the FLE antibody response to flavivirus infection, which may explain why even poor neutralizing FLE antibodies could protect mice from WNV lethal challenges [91].

Besides FLE antibodies, antibodies mapped to DI and DII hinge region were also found in natural infections of WNV and DENV [83,85,92]. Comparison of one of these antibodies, WNV CR4354, with hE16 showed that CR4354 inhibits WNV infection nearly as potently as hE16 and the neutralization occurs also at a post-attachment step similar to that of hE16 [92]. A cryo-EM structure of DENV1 complexed with the Fab of 1F4, a human mAb specific for DENV1, showed that 1F4 binds to the DI and the hinge of DI and DII within an E protein monomer [83]. Unlike CR4354, 1F4 not only inhibits infection at a post-attachment step, but also blocks viral attachment in a WNV receptor expressing cell line (Figure 1). Interestingly, a common feature is that both CR4353 and 1F4 bind to the intact virus but not the recombinant E protein. This indicates that these mAbs recognize a particular conformation of an epitope in the DI and DII hinge region that is preserved only in the intact virus [83,85,92]. Through screening of a large panel of naturally occurring human antibodies against DENV, Smith et al. identified a broadly neutralizing antibody 1C19 that recognizes a unique epitope on the BC loop of DII [82]. Surprisingly, 1C19 not only neutralizes all four DENV serotypes, but also competes with all weakly neutralizing FLE antibodies and 1F4 that are mapped to the DI/DII hinge region. This indicates that the BC loop is probably close enough to the DI/DII hinge on the intact virus so that the epitopes of 1C19 and 1F4 overlap with each other. This may be worth further exploring for antibody therapeutics development.

### 3.3. Antibodies that Recognize Quaternary Structures

Through screening of human mAbs from natural flavivirus infections, a new class of potent E protein-specific antibodies was identified. Surprisingly, epitope mapping could not assign these antibodies to a single E protein monomer. Instead, they bind to more complex quaternary structure epitopes on the E protein dimers [67,85,90,93,94,95,96,97]. Some of these antibodies are serotype specific [67,94,98] but others can be broadly neutralizing [90,96]. In general, these antibodies inhibit the E protein structural rearrangement during membrane fusion. However, some of them, such as HM14c10 and 5J7, neutralize the viral infection primarily through blocking viral attachment (Figure 1) [67,98].

The first reported mAb that binds to two adjacent E protein monomers was WNV CR4354 [99]. Subsequently, several DENV serotype-specific antibodies were mapped to the quaternary structure epitopes, such as anti-DENV1 mAb HM14c10 [67], anti-DENV2 mAb 2D22, and anti-DENV3 mAb 5J7 [85,94,98]. These mAbs showed potent neutralization activities against serotype-specific DENV infections. Similar to CR4354, these mAbs bind to regions between two adjacent E monomers, or in the case of 5J7, three E molecules [98]. These inter-monomer regions can be mapped to all three domains of the E protein depending on the specific mAb. Interestingly, a ZIKV-specific antibody (ZIKV-117) isolated from a ZIKV-infected patient, showed binding to DII of two neighboring dimers at the dimer-dimer interface [97,100]. The therapeutic efficacy of ZIKV-117 has been demonstrated in both pregnant and non-pregnant ZIKV mouse models. Another group of human antibodies isolated from DENV-infected patients, called “E-dimer-dependent epitope” (EDE)-specific antibodies, recognize a highly conserved site among all four serotypes at the E dimer interface, which is also the binding site for prM during virus maturation [101]. EDE-specific antibodies showed potent neutralization to all four DENV serotypes with 50% neutralization in low nM or even picomolar (pM) concentration range [90]. More excitingly, several of the EDE-specific antibodies were found to be protective against ZIKV infection in animal models [96,102]. For example, EDE1-B10 displayed strong cross-neutralization activity against multiple ZIKV strains and significantly reduced mortality in mice challenged with a lethal dose of ZIKV. Furthermore, the same mAb also reduced fetal demise in ZIKV infected pregnant mice [96]. These results support the feasibility of developing therapeutics against both ZIKV and DENV infections.

## 4. Antibody-Dependent Enhancement of Viral Infection

One of the most difficult hurdles in vaccine and antibody-based therapeutic development against flaviviruses is the risk of ADE. This phenomenon was first demonstrated in vitro and later in vivo for DENV pathogenesis that explains the development of severe dengue hemorrhagic fever (DHF) and dengue shock syndrome (DSS) upon secondary infection by a heterologous DENV serotype [106,107]. Since then ADE has been observed in a variety of viruses associated with serious human diseases [108]. More recently, human mAbs from patients previously infected by DENV have been shown to cross-react with ZIKV and cause ADE in ZIKV-infected FcγR-expressing cells [109,110,111,112]. Bardina et al. further demonstrated that human plasma containing anti-DENV or WNV antibodies could enhance clinical symptoms and increase mortality in a ZIKV mouse model [113]. Similarly, anti-ZIKV antibodies have been shown to enhance DENV infection in vivo [58]. These results have raised safety concerns for the development of vaccines and antibody therapeutics against flaviviruses as ZIKV is spreading in areas where DENV or WNV is endemic and mosquito species transmitting DENV are capable of transmitting ZIKV as well [114].

The most commonly known mechanism for ADE is that when viruses bind to non-neutralizing antibodies or antibodies at sub-neutralizing concentrations, the virus-antibody complex can also bind to the FcγR on myeloid cells (i.e., monocytes, dendritic cells, macrophages) through the antibody Fc region, thereby facilitating virus entry and increasing infectivity [108,115] (Figure 1). Alternatively, the complement activation pathways have also been reported in inducing ADE in a variety of viruses including flaviviruses [108,115]. Experiments with WNV and DENV showed that complement component C1q may restrict ADE in flavivirus infection in an IgG subclass-dependent manner [116] probably by reducing the stoichiometric threshold for neutralization [117]. A recent study suggested that E-specific antibodies may also induce ADE in a FcγR-independent manner by facilitating interactions between the flavivirus E protein fusion loop and lipids in the host cell membrane [118]. These findings highlight the complexity of ADE and may explain the discrepancy between laboratory data and clinical observations.

Because ADE may potentially magnify the severity of flavivirus infection, reducing ADE has been a major focus of antibody-based therapeutics development. One way of minimizing ADE is to design or screen for broadly and potently neutralizing antibodies. For example, the anti-DENV mAb ab513, which can potently neutralize all four serotypes of DENV, will have a low risk of ADE in enhancing any DENV infection [59]. However, ab513 still showed a certain degrees of ADE in an FcγR expressing cell line at sub-neutralizing concentrations. Nevertheless, ab513 is by far the most promising mAb-based therapeutic against DENV with a low risk of ADE when sufficient dosage would be administered to patients. 

L234A and L235A double mutations (LALA) in the IgG Fc domain have been shown to abrogate the binding of IgG to FcγRs [119]. The LALA variants have been found to share equivalent antigen-binding properties and neutralization potency as the WT antibodies [120,121]. Using this strategy, LALA variants of a few mAbs against ZIKV and DENV have been shown to have similar neutralizing potency as their WT equivalents, both in vitro and in vivo but without promoting ADE [94,96,100]. Although LALA mutations eliminate the risk of ADE, they also forego immune-mediated effector functions that may be important for the full efficacy of antibodies against viral infection [122,123]. For example, Hessell et al. showed that the LALA variant of a broadly neutralizing antibody against HIV dramatically decreased its ability to protect macaques from infection challenge due to the lack of ADCC activity [121]. Therefore, there is a critical need to develop mAb therapeutics against flaviviruses that forego ADE but retain their ability to fight infections via FcγR-mediated effector functions such as ADCC and CDC (Figure 1).

## 5. Plant-Produced Antibodies against Flaviviruses

### 5.1. Plants as a System for the Development and Production of Antibody-Based Therapeutics

Despite the development of aforementioned therapeutic candidates, the eventual approval of human therapeutics against flaviviruses may largely depend on (1) the elimination of the biosafety concern of ADE and (2) the speed and economics of antibody production. Due to the unique nature of plant expression systems, plants may provide solutions to overcome both the biosafety and economic challenges of flavivirus therapeutic development [124,125]. The current state-of-the-art platform for mAb production is based on mammalian cell cultures. While it has superb capabilities in producing high quality of mAbs, it requires heavy upfront capital investment and a long lead time to establish a mammalian culture facility [126]. In contrast, plant-based expression systems can generate large amounts of biomass for mAb production without the requirement of prohibitive capital investment for building fermentation facilities and the need of constructing expensive duplicate facilities for scaled-up production is also obviated [127,128]. Multiple techno-economic studies demonstrated that plant expression systems indeed can substantially reduce the production cost of protein biologics including mAbs, providing direct evidence to support the long-held belief that plants can lower the production cost of biologics [129,130]. Current FDA-approved mAb drugs are expensive, making them unaffordable for citizens of the majority of countries in the world [45]. Plant-produced mAbs will allow the production of mAb therapeutics affordable for people in the developing world, where the majority of serious flavivirus cases exist [131].

Innovations in plant expression vector development, particularly vectors for transient expression, have produced new plant expression systems with the flexibility and speed that cannot be matched by those based on mammalian cell culture [132,133,134,135]. For example, plant transient expression with “deconstructed” plant viral vectors allows the production of up to 5 mg of vaccines and mAbs per gram of leaf fresh weight (LFW) within 10 days of vector inoculation [132,135,136,137,138,139,140,141,142]. The rapid and high-level protein production capability of transient expression systems make them the optimal system to quickly and versatilely produce mAb-based therapeutics against flaviviruses such as DENV, WNV and ZIKV that have multiple lineages with unpredictable outbreaks in various parts of the world.

**Figure 3:**
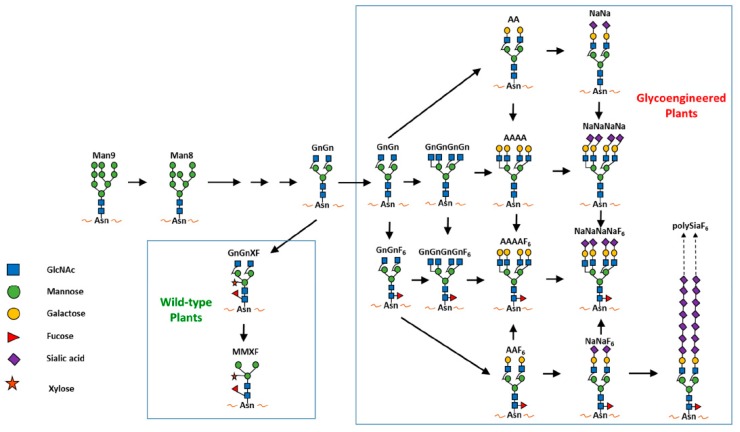
Major *N*-glycan structures produced in wild-type and glycoengineered plants [145].

Plants may also address ADE, the most difficult impediment of flavivirus therapeutic development. Since ADE relies on the interaction of the Fc region of antibodies with FcγRs, which is highly sensitive to the *N*-linked glycosylation pattern of the Fc region, the unique plant *N*-glycans may impact the properties of plant-produced mAbs, including ADE activities. In fact, the most exciting aspect of plant systems for mAb development is their amenability for glycoengineering. In contrast to mammalian cells, plants have a small repertoire of glycoenzymes. As a result, unlike mammalian cell-derived mAbs that exhibit a mixture of multiple *N*-glycans, plant-produced mAbs usually bear a single dominant *N*-glycan structure (Figure 3). Plant glycoproteins contain core α1,3-fucose and xylose that are not present in human glycoproteins in significant amounts [143]. These quantitative differences in *N*-glycan distribution between plant and human cells were a major concern, as plant-enriched glycans might trigger immune responses leading to production of plant-glycan specific antibodies that could reduce therapeutic efficacy or even cause adverse effects. However, all available reported results including both animal and human studies indicate that plant-derived *N*-glycans do not influence the overall immunogenic profile of plant-produced protein therapeutics [144]. Paradoxically, the small repertoire of glycoenzymes has benefited plants as hosts for developing mAbs with homogeneous human glycans [145]. In contrast, mammalian cells have a large glycome that impedes the manipulation of the *N*-glycosylation pathways [146]. By knocking out plant-specific glycan genes and introducing mammalian glycosylation genes, glycoengineering is able to generate plant hosts that produce mAbs with authentic human *N*-glycans with a degree of glycan homogeneity that cannot be produced by mammalian cells or by in vitro treatments [147,148,149]. This has silenced the concern that plant-derived mAbs would trigger the production of plant-specific antibodies in the host. This is due to the lack of any structural bases for inducing such immunity because these mAbs carry only genuine human glycoforms with the original amino acid backbone and any plant-specific impurities are eliminated by FDA-compliant downstream processing [143,145,150]. For example, a *Nicotiana benthamiana* line called ΔXF that does not produce plant-enriched *N*-glycans was created by suppressing the expression of two plant glycoenzymes [143]. A homogenous (>90%) GnGn *N*-glycan structure has been observed in various mAbs produced in ΔXF plants. These ΔXF plant-produced mAbs have also been shown to have significantly enhanced neutralization or ADCC potency [148,151]. The efficacy of ZMapp, a cocktail of three anti-Ebola mAbs produced in ΔXF plants, showcased the advantage of plant-produced mAbs. ZMapp was shown to have superior potency to their mammalian cell-produced counterparts and were able to rescue 100% of rhesus macaques even when given five days after a lethal Ebola challenge [152], leading to ZMapp’s compassionate use in human patients during the 2014 Ebola outbreak. The availability of a portfolio of plant lines that can produce biologics with tailor-made mammalian *N*-glycans on demand provides the opportunity to overcome efficacy and safety challenges against the development of mAb-based therapeutics including ADE. 

### 5.2. Plant-Produced Antibodies against WNV

Our laboratory has long been interested in the development of mAb-based therapeutics and vaccines against flaviviruses using plant expression systems. We first addressed the questions of if plants can produce mAbs robustly and if the plant-produced mAbs share similar properties and efficacy with their counterparts produced in mammalian cells, questions that were not answered at that time. We chose to use hE16 mAb against WNV as the test case. WNV can infect the CNS and lead to encephalitis and meningitis, with the elderly and immunocompromised at greatest risk. Over the last two decades, WNV has spread to the western hemisphere with more frequent outbreaks and increased cases of neuroinvasive diseases. Global WNV epidemics call for the development of more efficacious therapeutics and production platforms that can rapidly and inexpensively transfer effective therapeutics to the clinical setting. Our results showed that hE16 can be expressed at a very high level of 0.8 mg/g LFW within 8 days of infiltration in *N. benthamiana* plants with a transient expression system based on a tobacco mosaic virus-based deconstructed vector [103]. Furthermore, plant-produced hE16 (phE16) was detected to have identical binding affinity and kinetics for WNV E protein and DIII compared to hE16 produced in mammalian cells (mhE16) [103]. Our results also showed that phE16 and mhE16 also shared equivalent neutralization potency against WNV. Most importantly, a single-dose injection of phE16 protected mice from a lethal WNV challenge in both the pre- and post-exposure models; a result indistinguishable from that of mhE16 [103]. These findings are highly significant as they were the very first demonstration of post-exposure efficacy for a plant-produced mAb at that time. Downstream processing is an important component of a mAb production technology. We demonstrated that phE16 can be efficiently purified to homogeneity with a simple three-step extraction and purification scheme in a scalable and current Good Manufacture Practice (cGMP)-compliant manner [103]. To further investigate the feasibility of manufacturing plant-made mAbs in large scale, we explored lettuce as a host plant for producing hE16 [140]. Similar to tobacco plants, lettuce is already cultivated on a large scale commercially and can produce large quantities of biomass rapidly. Our study demonstrated that hE16 can be expressed and assembled as robustly and efficiently in lettuce as in *N. benthamiana* plants [140]. In fact, the highest level of hE16 accumulation occurred within four days of leaf infiltration [140], almost a week faster than in tobacco. Lettuce-produced phE16 has the same antigen-binding specificity and neutralization potency against WNV as mhE16. Significantly, phE16 can be purified to >95% homogeneity by a single protein A affinity chromatography step with levels of residual DNA, endotoxin and protein A below the FDA specifications for injectable mAb drugs [140]. This can be mostly attributed to the fact that lettuce plants produce negligible amounts of phenolics and alkaloids compared to tobacco plants. In fact, we demonstrated that direct loading of lettuce extract onto protein A resin did not foul the resin over 20 purification cycles. Therefore, this study demonstrated the feasibility of using commercially produced lettuce for high-level and rapid mAb production [140]. This allows our production system to have access to unlimited quantities of inexpensive plant material for industry-scale production. The robustness and scalability of the hE16 expression in lettuce, coupled with the simplified purification process and unlimited nature of plant material generation, provide a production platform for anti-flavivirus mAbs that is low-cost, safe, and amenable to large-scale manufacturing.

**Figure 4:**
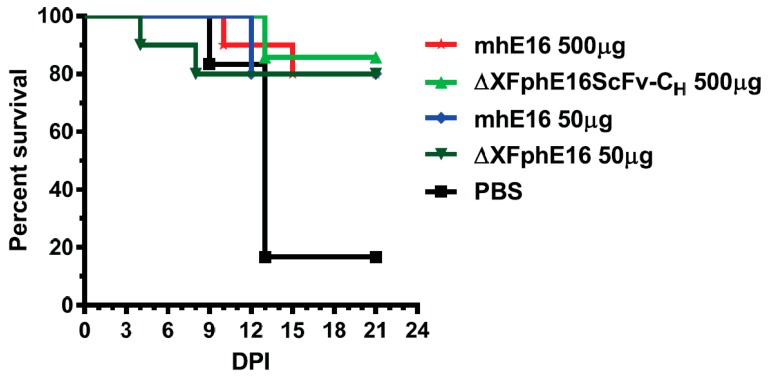
∆XFphE16 and ∆XFphE16scFv-C_H_ mediated protection in mice. Five week-old C57BL/6 mice were infected with 10^2^ PFU of WNV and then given a single dose of ∆XFpE16 (50 μg), ∆XFpE16scFv-C_H_ (500 μg) or mHu-E16 (50 μg or 500 μg) via an intraperitoneal route at day +4 after infection. Survival data from at least two independent experiments (*n* = 10 mice per dose) were analyzed by the log-rank test. (From [148] with permission from John Wiley and Sons).

To eliminate plant-enriched glycans and the risk of unnecessary immune responses, hE16 and a single chain variant E16scFv-CH were produced in the glycoengineered *N. benthamiana* line, ΔXF. Both ΔXF-produced mAbs (ΔXFphE16 and ΔXFphE16scFv-CH) displayed uniform mammalian-type *N*-glycosylation pattern of GnGn without the detection of residual plant-enriched glycans compared to the same mAbs expressed in WT *N. benthamiana* [148]. ∆XFhpE16 and ∆XFhpE16scFv-CH demonstrated equivalent antigen binding affinity and kinetics, and slightly enhanced neutralization of WNV compared to the mhE16. A single dose of ∆XFphE16 or ∆XFphE16scFv-CH protected mice against WNV-induced mortality, even four days after infection, at equivalent efficacy as mhE16 (Figure 4) [148]. Thus, this demonstrates the development of anti-WNV mAb therapeutic single-chain variants that are equivalent in efficacy to phE16, simpler and cheaper to produce, and likely safer to use as therapeutics due to their mammalian *N*-glycosylation.

**Figure 5:**
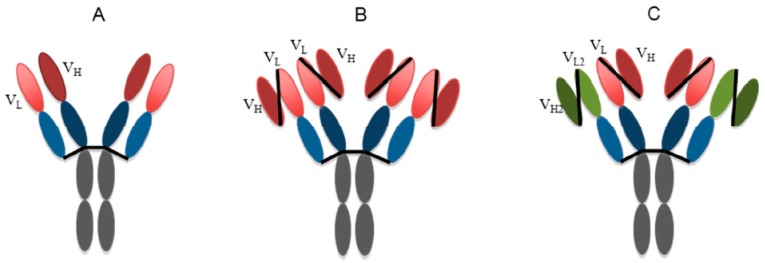
Tetravalent phE16 and bifunctional hE16 design. (**A**) phE16. (**B**) Tetravalent phE16. (**C**) Bifunctional phE16 that may have enhanced ability to cross the blood brain barrier while retaining the potency of neutralizing WNV. V_L_: variable region of light chain; V_H_: variable region of heavy chain; V_L2_/V_H2_: a second pair of antigen binding sites that may bind to receptors on endothelial cells of the BBB and facilitate the bifunctional antibody to cross the BBB via transcytosis.

WNV is a neurotropic virus and causes CNS infections. Even though phE16 (Figure 5A), ∆XFhpE16, and ∆XFhpE16scFv-CH have shown excellent efficacy, their window of clinical treatment will be limited when delivered through peripheral routes. These mAb-based molecules cannot pass the blood–brain barrier (BBB), thereby failing to accumulate in the brain in sufficient levels to neutralize WNV, which can efficiently enter the CNS. Therefore, it is desirable to develop hE16 variants that can cross the BBB more efficiently. With this in mind, we explored the design of a tetravalent molecule (Tetra phE16) assembled from hpE16scFv-CH with a second phE16scFv fused to the light chain (LC) constant region (Figure 5B) [153]. Our results indicated that Tetra phE16 was efficiently expressed and assembled in plants, despite its size and complexity. To assess the impact of differences in *N*-glycosylation on hE16 variant assembly and function, we expressed additional phE16 variants with various combinations of heavy chain (HC) and LC components, which revealed that proper pairing of HC and LC was essential for the complete *N*-glycan processing of antibodies in both plant and animal cells. Associated with its distinct *N*-glycoforms, Tetra phE16 displayed differential binding to C1q and various FcγRs. All plant-derived Tetra phE16 glycovariants showed at least equivalent in vitro neutralization and in vivo protection compared to mhE16. Excitingly, none of the plant-derived Tetra hE16 glycovariants had any ADE activity, alluding to the potential of plant-produced antibodies to minimize the adverse effect of ADE [153]. This study demonstrated the feasibility of producing large, complex and functional IgG-like tetravalent mAb variants in plants and also provided insight into the relationship between mAb *N*-glycosylation, FcγR and C1q binding, and ADE. The successful production and assembly of Tetra phE16 and the demonstration of its therapeutic activity brings us closer to developing bifunctional mAbs that can pass the BBB and have a longer window of efficacy. For example, bifunctional mAbs with a similar structure as the Tetra phE16 but with one of the two scFvs binding to a specific receptor (e.g., insulin receptor [154]) on the BBB may have the desired bi-functionality: one scFv would facilitate its transport into the brain and the other scFv would retain its therapeutic activity against WNV in the brain (Figure 5C).

Overall, these studies with phE16 and its variants demonstrated that plant-derived mAbs in various glycoforms can function effectively as post-exposure therapy against a potentially lethal flavivirus disease. Plants are an efficient platform to produce phE16, its single-chain, and tetravalent variants with high-yield, speed, enhanced scalability, and cost-effectiveness, satisfying all major metrics for successful therapeutic candidates. This technology can lead to safer, more efficacious therapeutic candidates, and be readily applied in the future to mAbs against other emerging flavivirus infections, and may be most useful in resource-poor settings such as the developing world.

### 5.3. Plant-Derived Antibodies against DENV and ZIKV

There are four serotypes of DENV (DENV1–4), and together, they represent one of the largest global disease burdens to date, with over 3 billion people at risk for infection and ~390 million infections in tropical and subtropical regions of the world annually [155]. DENV infections highlight the devastations that ADE may cause and the difficulty in developing mAb therapeutics and vaccines against flavivirus. Primary infection with one DENV serotype usually produces self-limiting Dengue fever (DF). However, secondary infection with another DENV serotype increases the risk of developing severe disease, including life-threatening vascular leakage syndrome, known as DHF/DSS [156,157]. The development of DHF/DSS in secondary infection is most likely caused by ADE as antibodies generated during a primary infection may be non-neutralizing or sub-neutralizing against a heterologous DENV serotype in a secondary infection [158]. Instead, these cross-reactive antibodies can enhance infection of the second DENV serotype in FcγR-expressing cells and lead to DHF/DSS [159] (Figure 1). The frequency and severity of DHF/DSS has increased significantly in recent years in regions that used to have outbreaks of mild DF [160,161]. This may be due to the geographic expansion of the DENV mosquito vectors and co-circulation of the four DENV serotypes in the same region promoted by global trade and international travel [162,163]. The risk of ADE severely hinders the development of mAb-based therapies for DENV because patients who are treated with mAbs against one serotype of DENV may be at risk to develop DHF/DSS through ADE if they are exposed to another serotype of DENV subsequently. Therefore, in order for mAbs to be effective therapeutics against DENV infection, their risk in inducing ADE needs to be eliminated.

Although it was previously reported that an aglycosylated anti-DENV mAb and LALA-backbone mAb mutants could eliminate the risk of ADE [94,96,100,164,165], the complete abolishment of binding to all FcγRs also may render the antibody unstable [166,167], and lose the necessary effector function for its full therapeutic efficacy [117,122,123,168,169]. Therefore, the optimal mAb-based therapeutics against DENV must be able to neutralize the virus and preferably retain the ability to induce ADCC and CDC, but not induce ADE.

Since the *N*-linked glycosylation of a mAb affects its FcγR and C1q binding, pharmacokinetics, effector function and efficacy [170], it is possible to identify specific mAb *N*-glycoforms that promote specific binding to a subset of FcγRs, which may eliminate ADE but retain ADCC and/or CDC activities of the mAb. Previous studies on this subject were scarce due to the difficulty in obtaining mAbs with a homogenous glycoform. Mammalian cells usually produce mAbs with glycan heterogeneity, even in glycoengineered cell lines [171]. The availability of glycoengineered plant lines that produce mAbs with various defined and uniform mammalian *N*-linked glycans provides a unique opportunity for us to evaluate the contribution of mAb carbohydrate moieties to Fc-mediated functions including ADE [143,145]. 

**Figure 6:**
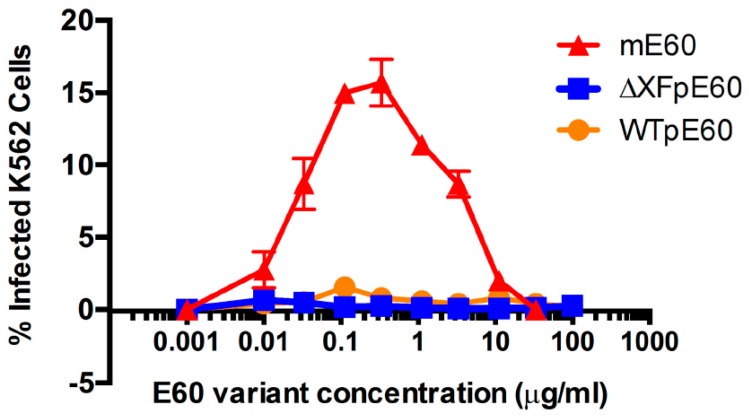
Antibody-dependent enhancement of pE60 variants. Serial dilutions of E60 variants were mixed with DENV-2 and added to FcγR-expressing K562 cells. Forty-eight hours later, cells were fixed, permeabilized and stained with anti-DENV E antibody 4G2 and analyzed by flow cytometry for DENV infection of cells. (From [104] with permission from JGV).

Our laboratory used E60 mAb as a model antibody to investigate if plants can help to reveal specific *N*-glycoforms that overcome ADE. E60 is a mAb that was found to be cross-reactive with the highly conserved fusion-loop in DENV E DII and efficiently neutralizes all four DENV serotypes [164,172]. As a result, E60 should have the potential to become a therapeutic with efficacy against all serotypes of DENV. However, E60 produced by mammalian cells (mE60) exhibits ADE during DENV infection both in vitro and in vivo [164]. As a result, mE60 has no therapeutic activity and may even render treated subjects more susceptible to develop life-threatening DHF/DSS in secondary infection [164]. We expressed E60 in both WT and ΔXF *N. benthamiana* plants. Our results demonstrated that E60 produced in WT (WTpE60) and ΔXF (ΔXFpE60) plants exhibited a single predominant expected *N*-glycoform with a high degree of homogeneity [104]. Furthermore, these E60 glycovariants retained specific binding to the E DII antigen with similar kinetics as mE60. WTpE60 and ∆XFpE60 also displayed neutralizing activity against multiple DENV serotypes with a potency similar to that of mE60 [104]. Most importantly, our results demonstrated that both WTpE60 and ∆XFpE60 forewent their ADE activity on FcγR-expressing K562 cells, in contrast to mE60 that exhibited strong ADE activity (Figure 6) [104]. Detailed *N*-glycan analysis indicates that WTpE60, ∆XFpE60 and mE60 displays GnGnXF_3_-GnGn-, and AAF_6_/AGnF_6_-type complex glycans, respectively [104]. This suggests that the α-1,6 fucose, the terminal β1,4-galactose (AA or AGn) or the combination of both in mE60 may be responsible for the induction of ADE. Our on-going in vivo studies suggest that E60 produced in plants may also have potent post-exposure therapeutic activities in protecting mice against lethal challenges of DENV in both infection only and ADE mouse models [173]. 

ZIKV is closely related to the four serotypes of DENV and its infection has been linked to the development of severe fetal abnormalities that include microcephaly and Guillain–Barré syndrome in adults [174,175,176]. In 2015, over 1.5 million people were infected with ZIKV in Brazil [177]. The recent ZIKV outbreaks further complicated the development of mAb therapeutics against flaviviruses. ZIKV and DENV will continue to co-circulate in many areas of the world because of their common mosquito vectors and geographical distributions. Importantly, antibodies against DENV and ZIKV have been found to enhance the replication of each other in vitro, strongly indicating ADE may occur between these two closely-related viral diseases [58,178]. As such, minimizing the ADE risk of heterologous flavivirus infection should be an important consideration for any ZIKV and DENV therapeutic development. Specifically, mAb therapeutics against DENV or ZIKV need to forego ADE, not only for heterologous serotypes/strains of the same virus, but their ADE activities should also be eliminated for the other closely related flavivirus. 

**Figure 7:**
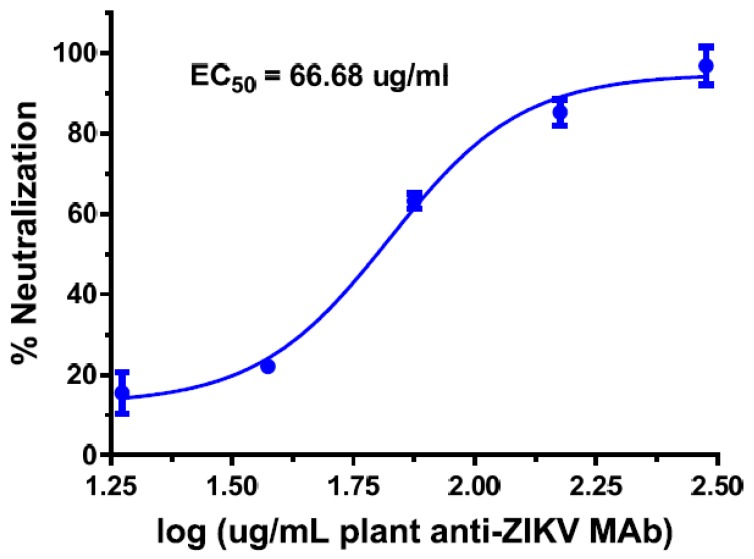
Neutralization of ZIKV by a plant-produced mAb. ZIKV (Puerto Rico strain) was incubated with serial dilutions of a ZIKV E protein-specific mAb and used to infect Vero cells. Cells were overlaid with fresh culture medium containing 0.8% agarose, fixed in paraformaldehyde, and then stained with crystal violet. EC50 value is listed in the graph.

In response, our laboratory has developed several mAbs against both ZIKV E DIII and DI-DII epitopes. When these anti-ZIKV mAbs were expressed in *N. benthamiana* plants, they can be produced and assembled efficiently. Moreover, some of them have potent neutralizing activity against ZIKV as well as DENV (Figure 7). We also produced these mAbs in mammalian cells as controls. The comparison between anti-ZIKV mAbs produced in mammalian cells and plants revealed that glycovariants of plant-made mAbs had drastically reduced ADE activity in enhancing DENV infection in contrast to their mammalian-cell counterparts [179]. Likewise, plant-produced anti-DENV E60 glycovariants also forwent their ADE activity for ZIKV infection [173]. Of note, in contrast to an aglycosylated mAb or LALA mutant, plant-produced glycovariants carry *N*-glycans that bind C1q and a subset of FcγRs, potentially preserving CDC and ADCC activity. Therefore, our study demonstrates that plant mAbs may be a preferred therapeutic candidate against DENV and ZIKV compared to mammalian cell-produced, aglycosylated or LALA mutants, as mAbs produced in mammalian cells induce ADE, and aglycosylated or LALA mAb mutants lose both ADCC and CDC activity and may have a shorter half-life in circulation. Our study provides so far unknown insight into the relationship between mAb *N*-glycosylation and ADE, which contributes to our understanding of how sugar moieties of antibodies modulate Fc-mediated functions and viral pathogenesis. Although further elucidation of the precise mechanisms of ADE abatement is warranted, our results will have important implications for mAb therapeutics beyond the DENV and ZIKV models. Thus, the ability to potentially eliminate ADE by plant-produced mAbs will lead to the development of safer and more efficacious antibody-based therapeutics against other ADE-prone viruses such as coronaviruses, paramyxoviruses, and lentiviruses [180].

## 6. Conclusions

The expanding global epidemics of flaviviruses ignite the urgent development of therapeutics against these devastating pathogens. MAb-based therapies have shown promise in providing specific and effective treatments against several flaviviruses including WNV, DENV and ZIKV. However, there is still no licensed human therapeutics for treating any flavivirus infections. Obstacles blocking the approval of mAb drugs against flavivirus include issues related to drug safety, economics and the speed of drug production. The risk of ADE in enhancing the severity of symptoms during secondary infection by a heterologous flavivirus in mAb-treated patients presents the most serious impediment for mAb therapeutic development. Flaviviruses such as DENV, WNV and ZIKV have multiple serotypes, strains and lineages with unpredictable outbreaks in various parts of the world. This requires a mAb production platform that is versatile and can rapidly produce anti-flavivirus mAbs on a large scale. The cost of producing mAbs is another major issue for the realistic implementation of treatment programs in the developing world, where the majority of flavivirus cases exists. While mammalian cell-produced mAbs are superb in many aspects, they are expensive, slow to produce, and have been shown to have strong ADE activity. In contrast, research by our laboratory and others have demonstrated that plant-based expression systems are able to rapidly and robustly produce high levels of anti-flavivirus mAbs at a significantly lower cost on a large scale, addressing both the versatility and economic issues of the mammalian expression systems. More importantly, we have demonstrated that plant-produced mAb glycovariants forego ADE while maintaining potent neutralizing and therapeutic activities against several important flaviviruses, and potentially retaining ADCC and CDC activities. We speculate that plant-based systems will facilitate the development of efficacious, safer, and affordable mAb therapeutics against flaviviruses and their eventual licensure and commercial production.

## Figures and Tables

**Table 1:** Flavivirus antibodies with therapeutic potential.

Antibody Name	Target Virus	Epitope	Development Stage	References
hE16, pE16	WNV	Lateral ridge of DIII	Phase II trial	[57,66,103]
CR4374	WNV	E protein DIII	Preclinical/mouse model	[77]
CR4354	WNV	E protein Hinge between DI, DII	Preclinical/mouse model	[77,92]
CR4348	WNV	E protein DII	Preclinical/mouse model	[77,92]
Plant-made E60	DENV1–4	E protein DII fusion loop	Preclinical/mouse model	[104]
DENV1-E105, E106	DENV1	E protein DIII lateral ridge	Preclinical/mouse model	[69]
1F4	DENV1	E protein DI, DI-DII hinge	Preclinical/mouse model	[83]
1C19	DENV1–4	E protein DII BC loop	Preclinical/mouse model	[11,82]
HM14c10	DENV1	E protein dimer-dimer interface	Preclinical/mouse model	[67,95]
2D22	DENV2	E protein dimer-dimer interface	Preclinical/mouse model	[85,93,94]
5J7	DENV3	Across three E protein	Preclinical/mouse model	[98,105]
Ab513	DENV1–4	E protein DIII	Preclinical/mouse model	[59]
2A10G6	DENV1–4, WNV, Zika, YFV, TBEV	E protein fusion loop	Preclinical/mouse model	[54,84]
ZKA64	Zika	E protein DIII	Preclinical/mouse model	[58]
ZV54	Zika	E protein DIII Lateral Ridge	Preclinical/mouse model	[61]
ZV67	Zika	E protein DIII Lateral Ridge	Preclinical/mouse model	[61]
Z23	Zika	E protein DIII	Preclinical/mouse model	[81]
Z3L1	Zika	E protein DI, DII	Preclinical/mouse model	[81]
Z004	Zika/DENV1	Lateral Ridge in DIII	Preclinical/mouse model	[60]
ZIKV-117	Zika	E protein dimer-dimer interface	Preclinical/mouse model	[97,100]
EDE1-B10	Dengue/Zika	E protein dimer-dimer interface	Preclinical/mouse model	[96]
